# Characterization of Protein Phosphatase 5 from Three Lepidopteran Insects: *Helicoverpa armigera*, *Mythimna separata* and *Plutella xylostella*

**DOI:** 10.1371/journal.pone.0097437

**Published:** 2014-05-13

**Authors:** Xi’en Chen, Shumin Lü, Yalin Zhang

**Affiliations:** Key Laboratory of Plant Protection Resources & Pest Management of the Ministry of Education, Northwest A&F University, Yangling, Shaanxi, China; Natural Resources Canada, Canada

## Abstract

Protein phosphatase 5 (PP5), a unique member of serine/threonine phosphatases, regulates a variety of biological processes. We obtained full-length PP5 cDNAs from three lepidopteran insects, *Helicoverpa armigera*, *Mythimna separata* and *Plutella xylostella*, encoding predicted proteins of 490 (55.98 kDa), 490 (55.82 kDa) and 491 (56.07 kDa) amino acids, respectively. These sequences shared a high identity with other insect PP5s and contained the TPR (tetratricopeptide repeat) domains at N-terminal regions and highly conserved C-terminal catalytic domains. Tissue- and stage-specific expression pattern analyses revealed these three PP5 genes were constitutively expressed in all stages and in tested tissues with predominant transcription occurring at the egg and adult stages. Activities of *Escherichia coli-*produced recombinant PP5 proteins could be enhanced by almost 2-fold by a known PP5 activator: arachidonic acid. Kinetic parameters of three recombinant proteins against substrate pNPP were similar both in the absence or presence of arachidonic acid. Protein phosphatases inhibitors, okadaic acid, cantharidin, and endothall strongly impeded the activities of the three recombinant PP5 proteins, as well as exerted an inhibitory effect on crude protein phosphatases extractions from these three insects. In summary, lepidopteran PP5s share similar characteristics and are all sensitive to the protein phosphatases inhibitors. Our results also imply protein phosphatase inhibitors might be used in the management of lepidopteran pests.

## Introduction

Reversible phosphorylation of proteins accomplished by opposing functions of protein phosphatases and protein kinases is believed to affect a diverse array of cellular activities [1]. Eukaryotic serine/threonine phosphatases are structurally and functionally diverse enzymes that are represented by three major families: phosphoprotein phosphatases (PPP), metal-dependent protein phosphatases (PPM), and the aspartate-based phosphatases represented by FCP/SCP. Representative members of the PPP family comprise the signature phosphatases, PP1, PP2A, and PP2B (or calcineurin), as well as the recently discovered phosphatases, such as PP4, PP5, PP6, and PP7. The PPM family includes the Mg^2+^-dependent phosphatases, such as PP2C and pyruvate dehydrogenase phosphatase [2], [3].

PP5 is widely expressed but at a lower level than most other serine/threonine protein phosphatases in mammals, and it is highly conserved among other eukaryotes [2], [3]. Unlike most other phosphatases having isoforms encoded by different genes, the regulatory and catalytic domains of PP5 are combined within the same chain. PP5 contains three tetratricopeptide repeat (TPR) motifs at its N-terminus which serve as protein-protein interaction motifs. Removal of the TPR domains by limited proteolysis leads to a substantially elevated phosphatase activity [4], and recent structural analyses indicate that the access to the active site of the phosphatase is blocked by TPR domains [5]. Moreover, TPR domains are also involved in the stimulation of PP5 activity via interaction with polyunsaturated fatty acids [4], [6]. Mammalian PP5 has been found to play an important role in hormone and stress induced signaling, DNA repair, intracellular proliferation, differentiation, migration, survival and death [7]–[9]. However, knowledge about PP5 in insects is poorly known.

In this study, we identified PP5 genes from three lepidopteran insects, *H. armigera*, *M. separata* and *P. xylostella*, and analyzed their expression. Recombinant PP5 proteins were produced in *E. coli* and purified. Their biochemical properties and sensibility to inhibitors were also investigated.

## Materials and Methods

### Insects and Chemicals

An insecticide-susceptible strain of *P. xylostella,* maintained in the laboratory for >5 yr without exposure to insecticides, was reared on pakchoi cabbage at 25±2°C, 50±5% relative humidity and a photoperiod of 16L:8D. *H. armigera* larvae were procured from Zhongke Baiyun (Beijing, China) and reared on artificial diets at 25±2°C, 50±5% relative humidity and a photoperiod of 14L:10D. *M. separata* were initially obtained from a culture at the Biorational Pesticides Research and Development Center, Northwest A&F University, Shaanxi, China and reared on corn leaves at 25±2°C, 50±5% relative humidity and a photoperiod of 14L:10D. All moths were supplied with a 5% honey solution as nutrient and permitted to oviposition on moist gauze sterilized with a 1% sodium hypochlorite solution gauze.

Okadaic acid, cantharidin, arachidonic acid, and pNPP were purchased form Sigma (St. Louis, Missouri, USA). Endothall was purchased from Alfa Aesar (Haverhill, MA, USA). All other chemicals were of research grade or better and were obtained from commercial sources.

### Cloning and Sequences Analysis of PP5 Genes

Total RNA was extracted from the larvae of the 4th, 5th, and 6th instar larvae of *P. xylostella*, *H. armigera*, and *M. separata*, respectively, using Trizol plus (TaKaRa, Dalian, China) according to manual instructions, and treated with DNase using a DNase I kit (Fermentas, Ontario, Canada). Then cDNA was synthesized using a First Strand cDNA Synthesis kit (Fermentas, Ontario, Canada). Degenerate primers were designed based on the PP5 amino acid sequences from *Bombyx mori* (XP_004923376), *Danaus plexippus* (EHJ67807), *Tribolium castaneum* (XP_971407), *Dendroctonus ponderosae* (AEE62915), *Megachile rotundata* (XP_003699533), *Nasonia vitripennis* (XP_001603324), *Apis mellifera* (XP_624242), *Drosophila willistoni* (XP_002070260) and *Aedes aegypti* (XP_001650298), by using two online tools, Blocks WWW Server (http://blocks.fhcrc.org/blocks/) and CODEHOP (http://bioinformatics.weizmann.ac.il/blocks/codehop.html). A pair of primers was used in PCR with first strand cDNA as the template under the following PCR conditions: 94°C for 3 min, 35 cycles of 94°C for 30 s, 60°C for 30 s, 72°C for 1.5 min, followed by a final extension at 72°C for 7 min. The purified fragment was inserted into pMD19-T vector (TaKaRa, Dalian, China) and transformed into *E. coli* DH5α (TaKaRa, Dalian, China), then sequenced (AuGCT, Inc., Beijing, China).

Rapid amplification of cDNA ends (RACE) was conducted using the 5′-Full RACE kit (TaKaRa, Dalian, China) following manual instructions. RACE specific primers were designed from the obtained fragments. PCR conditions were as follows: 94°C for 3 min, 35 cycles of 94°C for 30 s, 60°C for 30 s, 72°C for 2 min, and final extension at 72°C for 7 min. PCR products were sequenced as mentioned above. The open reading frame (ORF) was predicted using ORF Finder (http://www.ncbi.nlm.nih.gov/gorf/gorf.html). Primers used to amplify the ORFs were designed using Primer Premier 5 and were incorporated with a *Nde I* restriction site at 5′ends of forward ones, while a *Hind III* restriction site at 5′ends of reverse ones. PCR conditions were as follows: 94°C for 3 min, 35 cycles of 94°C for 30 s, 62°C for 30 s, 72°C for 2 min, followed by a final extension at 72°C for 7 min. PCR products were sequenced as mentioned above. All primers used in this study were listed in Table 1.

**Table 1:** Sequences of all primers used in this work.

Gene	Primer name	sequence (5′–3′)
	Conversed region-forward	CACATGTCCCTGGGCAAGtwyaarytngc
	Conversed region-reverse	GGCGGAGAACACGGTGatrcayttncc
HaPP5	HaPP5-5′Race-forward	CCAGAGACGGTTGCTTTTGTAG
	HaPP5-5′Race-reverse	ATCCGCTATGTTGACCTCCTTC
	HaPP5-3′Race-forward	GAAACCACGAAACCCTGGACAT
	HaPP5-3′Race-reverse	GTGGACAGGAATAAGCAGCCGC
	HaPP5-ORF-forward	CATATGGCTACTAATGAAGAAATTAC
	HaPP5-ORF-reverse	AAGCTTTCATTGGCACAGCATGTTGAA
	HaPP5-qPCR-forward	ACGGTGGTCTGTTCTCCAAG
	HaPP5-qPCR-reverse	CCCATCGTTCTTGACCTCAT
MsPP5	MsPP5-5′Race-forward	TGACCACCCCATCTTCAAGTGC
	MsPP5-5′Race-reverse	CAACGGAGATGGCCTTCTCAAAG
	MsPP5-3′Race-forward	AAGCAGCCGCCTGAAGATGGTA
	MsPP5-3′Race-reverse	CACGAGGTCAAGAACGACGGCT
	MsPP5-ORF-forward	CATATGGCTAGTAACGAAGAAATTAC
	MsPP5-ORF-reverse	AAGCTTTCATTGGCACAACATGTTGAA
	MsPP5-qPCR-forward	TTTGTCACCGAGTTGATGGA
	MsPP5-qPCR-reverse	GATGTCTCCGCAGACTGTGA
PxPP5	PxPP5-ORF-forward	CATATGGCAAGTAACGATGAAATTACC
	PxPP5-ORF-reverse	AAGCTTCTACTGACAGAGCATGCTGAG
	PxPP5-qPCR-forward	CCTGCAAAAGCAACCTTCTC
	PxPP5-qPCR-reverse	CGGTCCACAAAGTCTCCATT
HaEF-1α	HaEF-1α-qPCR-forward	GCCTGGTACCATTGTCGTCT
	HaEF-1α-qPCR-reverse	ATCCGTTTGAGATTTGACCG
MsActin	MsActin-qPCR-forward	AGCTCTGCTACGTCGCTCTC
	MsActin-qPCR-reverse	CAAGCTTCCATACCCAGGAA
PxActin	PxActin-qPCR-forward	GCGACTTGACCGACTACC
	PxActin-qPCR-reverse	GGAATGAGGGCTGGAACA

The similarity analysis of deduced amino acid sequences was performed using BLAST programs (http://www.ncbi.nlm.gov/blast/). The phylogenetic analyses were conducted by MEGA 5.0 by the neighbor-joining method with bootstrap test of 1,000 replicates using amino acid sequences of other insect PP5s and the human PP5 (NP_006238) as an outgroup. The ExPASy Compute pI/Mw tool (http://web.expasy.org/compute_pi/) was used to predict the molecular weight and isoelectric points of PP5s. For the analysis of protein domains, the InterProScan 4 from EMBL-EBI (http://www.ebi.ac.uk/Tools/pfa/iprscan/) was used to search the InterPro collection of protein signature databases.

### Stage- and Tissue-dependent Expression Analyses

To study stage-specific expression, the eggs, 1st–4th instar larvae, pupae and adults of *P. xylostella*, and the eggs, 1st–6th instar larvae, pupae and adults of *H. armigera* and *M. separata* were collected and stored at −80°C until use. For tissue-specific expression study, four tissues including the head, gut, body wall muscle, and haemolymph from *P. xylostella* 4th larvae; six tissues containing the head, midgut, Malpighian tubule, accessory gland, ovary, and testis from *M. separata* adults; and six tissues comprising head, midgut, Malpighian tubule, trachea, fat body, and body wall muscle from *H. armigera* 6th instar larvae were dissected on ice, respectively. These were then snap frozen and stored at −80°C until use. Total RNA was extracted as described above. cDNA was synthesised from 1.0 µg total RNA using a PrimeScript RT reagent kit with gDNA eraser (TaKaRa, Dalian, China). Primers were designed using an online tool, Primer 3 (http://www.simgene.com/Primer3) (Table 1). The *P. xylostella* actin gene (accession: JN410820), *H. armigera* elongation factor-1α (EF-1α) gene (accession: U20129), and *M. separata* actin gene (accession: GQ856238), were used as references. Using 100-fold diluted cDNAs as templates, the Real-time quantitative PCR (qPCR) reactions were carried out on a thermal cycler (iQ 5, Bio-Rad, Philadelphia, PA, USA) using the Ultra SYBR Mixture (CWBIO, Beijing, China) according to the manual instructions. Thermal cycling conditions were 95°C for 10 min, 40 cycles of 95°C for 15 s, and 60°C for 1 min, then followed by dissociation analysis to check the homogeneity of the PCR product. The qPCR was repeated three times for each gene. Each replicate was performed with an independent RNA sample preparation and consisted of three technical replicates. Relative transcript levels of PP5 genes were determined by the 2^−Δ*Ct*^ equation: 2^−[*Ct*tar−*Ct*ref]^ (*Ct*tar, *Ct* value of PP5 genes; *Ct*ref, *Ct* value of reference genes). The expression data were presented as means ± standard deviation (SD) and were analyzed by One-Way ANOVA.

### *E. coli* Expression and Purification of Recombinant PP5

The open reading frames (ORF) of PP5 with *Nde I* site at N-terminal, and *Hind III* site at C-terminal, were cloned into the corresponding sites of pET28a (+) expression vector (Novagen, Madison, WI, USA), and transformed into *E. coli* BL21(DE3)plysS cells (Novagen, Madison, WI, USA). The positive transformed cells were amplified in LB medium containing 4 mM Mn^2+^, 34 µg ml^−1^ chloramphenicol and 100 µg ml^−1^ kanamycin. Recombinant PP5 expression was induced with 0.1 mM isopropyl β-D-thiogalactoside (IPTG) when *A*_600_ reached 0.4–0.6 and incubated for 48 h at 18°C.

Cells were harvested by centrifugation at 10,000 g for 20 min, 4°C and sonication for 5 min on ice in Buffer A (20 mM Tris-HCl, pH = 8.0, 300 mM NaCl, 4 mM MnCl_2_) comprising 0.1% β-mercaptoethanol, 1.0 mg ml^−1^ iysozyme and 1 mM PMSF. After centrifugation for 20 min at 20,000 g, 4°C, the soluble fraction was loaded onto a Buffer A pre-equilibrated Ni-NTA affinity column (Transgen, Beijing, China) to bind the target protein and the non-target proteins were washed down with Buffer A containing 20 mM imidazole. The target protein was eluted with 5 column volumes of Buffer A containing 150 mM imidazole. Eluted solutions were subjected to 12% SDS-PAGE and stained with Coomassie Blue. The high purity fractions were pooled and dialyzed against Buffer B (Buffer A +50% glycerol) overnight and stored at −20°C. Protein concentration was assessed using the method of Bradford [10].

### Crude PPP Extraction

One gram of 4th instar larvae of each insects were ground into a homogeneous state in ice-cold 20 mM Tris (pH = 7.5), 0.1% β-mercaptoethanol, 1 mM EDTA, 1 mM benzamidine and 1 mM (PMSF). This was then centrifuged at 12,000 g for 30 min at 4°C. The supernatant extract was desalted using Sephadex G-25 spin columns (GE Healthcare, Boston, MA, USA) to remove the contaminating free phosphate as described in the instruction manual. Each insect extract contained three biological replicates. Protein concentration was estimated using the method of Bradford [10].

### Determination of Phosphatase Activity

Activity of each recombinant PP5 against the inorganic phosphatase substrate pNPP was determined as described with slight modifications [11]. Assays were performed at 30°C in a 100 µL reaction containing 500 ng purified protein, 150 µM arachidonic acid and 20 mM pNPP in 20 mM Tris (pH = 7.5), 1 mM EDTA, 1 mM EGTA, 0.1% β-mercaptoethanol and 0.1% ethanol. After being pre-warmed to 30°C, the reaction was initiated by adding the pNPP. Assays were terminated after 15 min with 100 µL of 5 N NaOH. Non-enzyme samples were taken as controls. Sample absorbance was measured at *A*_410_ on an Infinite 200 PRO multimode micro-plate reader (Tecan, Austria). Kinetic parameters of each recombinant PP5 were estimated by using a Michaelis-Menten plot analysis of data obtained under the above assay conditions with pNPP concentrations of 1, 2, 5, 10, 20, 40, and 80 mM.

Phosphatases activity of crude extract was determined towards phosphopeptides using PPP assay kit (Promega, Madison, WI, USA) in half-area 96-well plates (Corning, NY, USA) following the manual instruction. In brief, 1.0 µg protein was mixed in assay buffer of 50 mM imidazole (pH 7.2), 0.2 mM EGTA, 0.02% β-mercaptoethanol, 0.1 mg ml^−1^ BSA, and pre-warmed to 30°C. Reaction was initiated after adding 100 µM phosphopeptides, RRA(pT)VA to obtain a final volume of 50 µL and incubated at 30°C for 15 min. The reaction was stopped by adding 50 µL of molybdate dye/additive mixture and incubated for an additional 15 min at room temperature for color development. Control samples were determined without enzyme. Sample absorbance was measured at *A*_600_ on an Infinite 200 PRO multimode micro-plate reader (Tecan, Austria).

Using pNPP as substrate, the optimum reaction temperature of recombinant PP5 protein was assayed at different temperatures and its optimum reaction pH was determined in various pH substation solutions. All assays above were performed with three replications.

### Inhibition Assay

Three candidate inhibitors were dissolved in dimethyl sulphoxide (DMSO) to provide stock solutions of 10 mM for okadaic acid, 100 mM for cantharidin, and 200 mM for endothall, respectively. All solutions were diluted to the desired concentrations with assay buffer before use. Assays were carried out using pNPP as substrate for purified proteins or using RRA(pT)VA as substrate for crude PPP extract. Before initiating the reaction, the enzyme was incubated with the chemicals for 5 min at room temperature. A dose-response assay was used to determine the IC_50_ values. The non-enzyme reaction was taken as the background control, while the non-inhibitor reaction was used as the full-activity control.

## Results and Discussion

### Cloning and Sequence Analysis

Although PP5 genes from several insect species have been identified and are available on NCBI Database, the information of their sequences characterization and their functional roles in biological processes is limited. In our previous work, a full-length PP5 cDNA was cloned from *P. xylostella* (accession: KF255601) [12]. By performing RT-PCR and RACE, we cloned full-length PP5 cDNAs from *H. armigera* and *M. separata.* Analysis of these three PP5 cDNAs revealed that cDNA of PxPP5 has a 5′ UTR of 102 bp, a 3′ UTR of 620 bp, and an open reading frame (ORF) of 1476 bp that encodes a protein of 491aa, with the theoretical pI of 6.54 and calculated molecular weight of 56073.10 Da; HaPP5 cDNA owns a 5′ UTR of 118 bp, a 3′ UTR of 396 bp, and an ORF of 1473 bp, encoding a protein of 490 aa, with the theoretical pI of 6.20 and calculated molecular weight of 55967.92 Da; while MsPP5 cDNA possesses an 1473 bp ORF which encodes a 490 aa protein, with theoretical pI of 6.20 and calculated molecular weight of 55824.71 Da, and the lengths of 5′ UTR and 3′ UTR are 118 bp and 396 bp, respectively. The full-length cDNAs of HaPP5 and MsPP5 were deposited into the GenBank Database with the accession numbers of KF983016 and KF983017, respectively.

**Figure 1:**
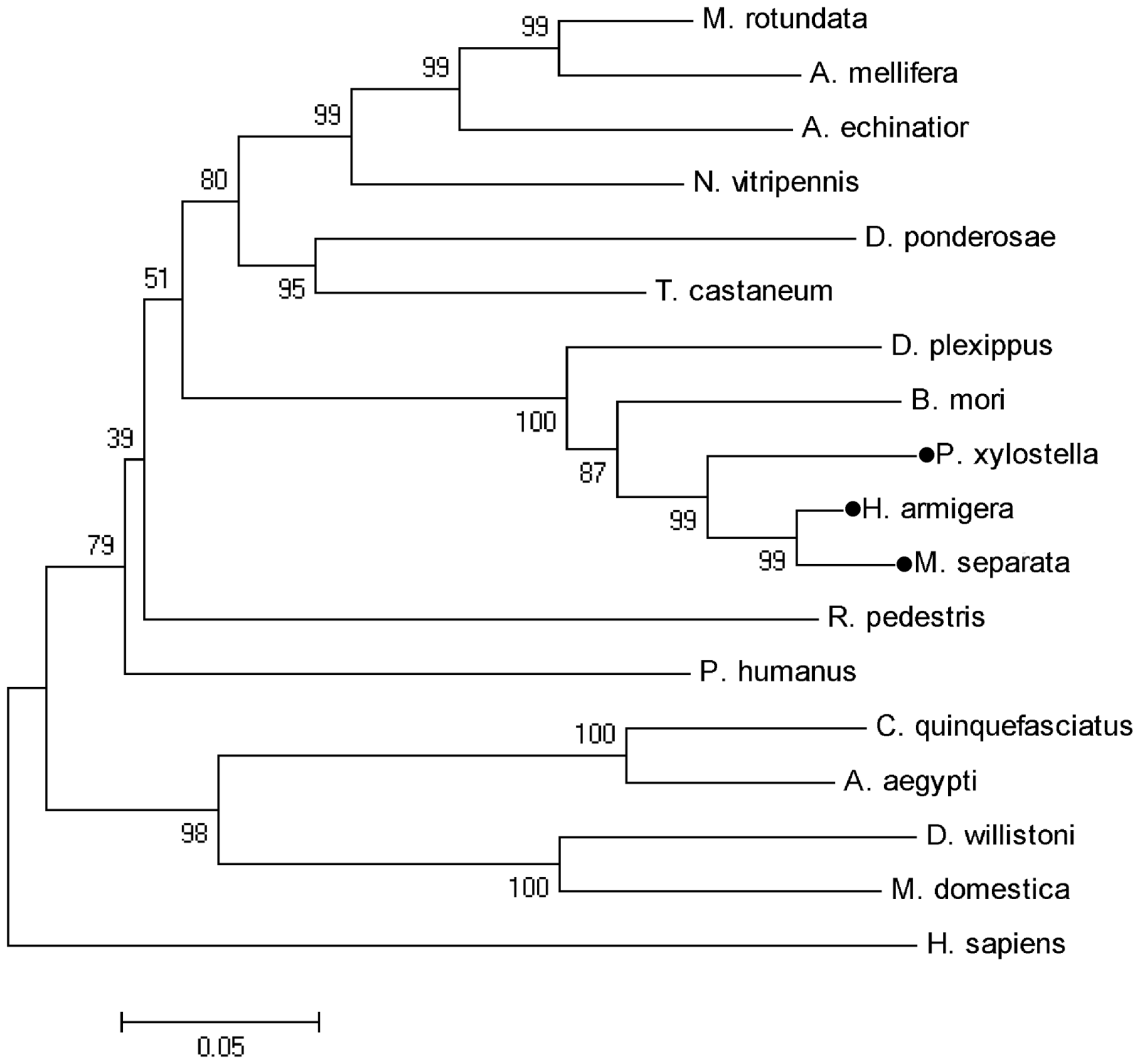
Phylogenetic relationships of HaPP5, MsPP5, and PxPP5 with other insect PP5s. Nodes with distance bootstrap values (1000 replicates) are shown. HaPP5, MsPP5, and PxPP5 are marked with filled circles.

**Figure 2:**
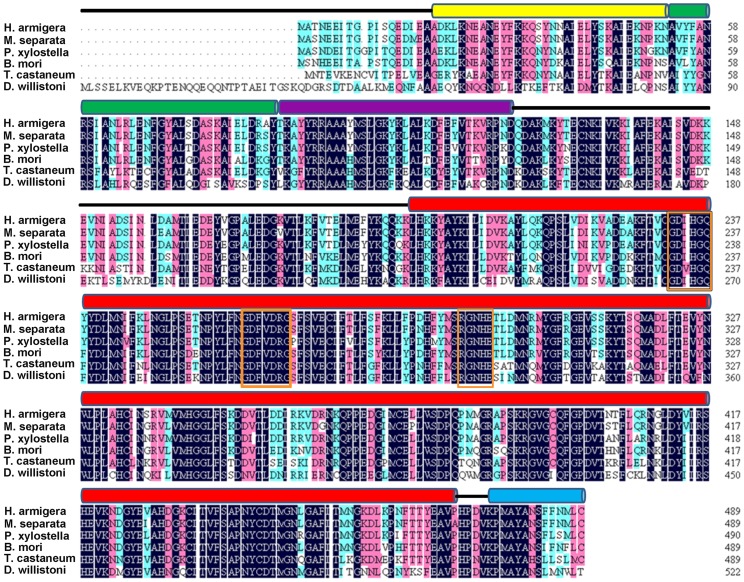
Amino acid sequence comparison of insect PP5s. Alignment of the deduced amino acid sequences of HaPP5, MsPP5, and PxPP5 were made with other insect PP5s from *B. mori* (XP_004923376), *T. castaneum* (XP_971407), and *D. willistoni* (XP_002070260). The positions of the three TPR (tetratricopeptide repeat) domains are indicated with lines of yellow, green, and purple above the sequences. The catalytic domain and helix αJ motif are indicated with a red line and blue line above those sequences, respectively. Three characteristic motifs are marked with orange rectangles.

BLAST analysis of deduced amino acid sequences of these three PP5 genes on NCBI revealed their strong similarities with PP5s from other insect species. They also shared >60% identity to human PP5. The constructed phylogenetic tree showed PP5 from lepidopteran insects form a small cluster (Fig. 1). Prosite analysis of obtained PP5 amino acid sequences revealed the presence of three TPR motifs at their N-terminus (Fig. 2). The C-terminal half of proteins consist of the highly conserved phosphatase domain containing three characteristic signature motifs within Ser/Thr phosphatases, GDxHG, GDxVDRG, and GNHE, which have been demonstrated to play important role in metal coordination, substrate binding and catalysis [13], . The helix αJ motif, which could strengthen the association between the TPR domains and the phosphatase domain of PP5 (Yang et al. 2005), was also present in their C-terminus.

### Tissue- and Stage-specific Expression Patterns

**Figure 3:**
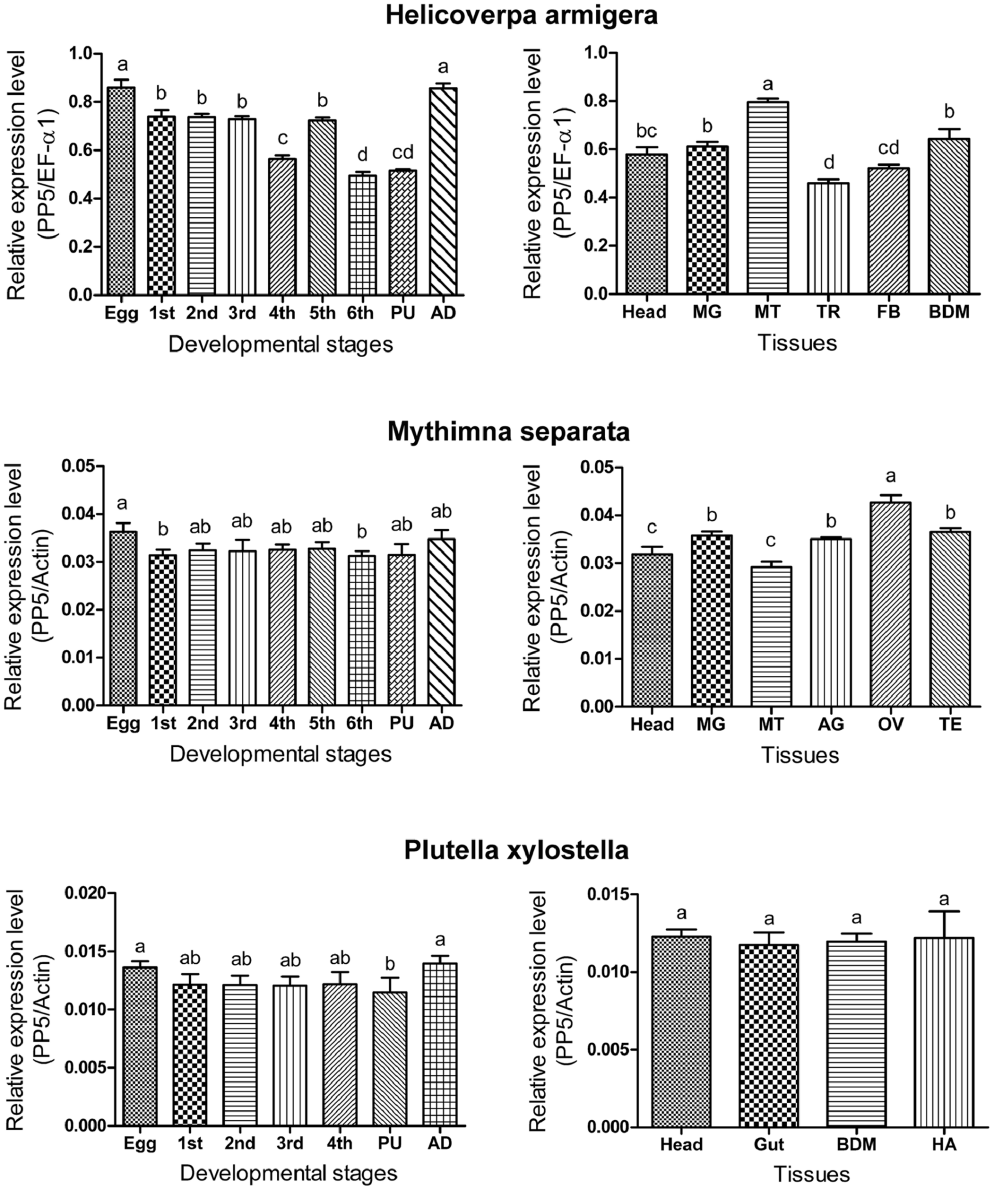
Relative mRNA expression levels of HaPP5, MsPP5, and PxPP5 in different developmental stages and various tissues. Abbreviation: 1st, first instar larvae; 2nd, second instar larvae; 3rd, third instar larvae; 4th, fourth instar larvae; 5th, fifth instar larvae; 6th, sixth instar larvae; PU, pupae; AD, adult; MG, midgut; MT, Malpighian tubule; TR, trachea; FB, fat body; BDM, body wall muscle; AG, accessory gland; OV, ovary; TE, testis; HA, haemolymph. The statistic differences were tested by One-Way ANOVA.

Stage-specific expression patterns of the PP5 gene were determined in all life stages of *H. armigera*, *M. separata*, and *P. xylostella* by qPCR reactions. Our results showed that PP5 genes were expressed in all stages of the three insects. Expression levels of the HaPP5 gene were relatively high in the egg and adult stages but significantly lower in the 4th instar, 6th instar, and pupa stages. MsPP5 gene transcripts had the lowest expression at the 6th instar stage and the highest expression in the egg stage. The PxPP5 gene was more abundantly expressed in the egg and adult stages, while it showed the lowest expression level in the pupa stage (Fig. 3).

Analysis of tissue-specific expression profiles of three insect PP5 genes revealed their widespread expression in various tissues. The PxPP5 gene was almost equally expressed in the four tested tissues, while transcript levels of the MsPP5 gene and HaPP5 gene were high in Malpighian tubules and the ovary, respectively (Fig. 3).

In *D. melanogaster*, PP5 was expressed across the life cycle, but more highly expressed in the embryonic than later developmental stages [15]. In *Epicauta chinensis*, PP5 was also constitutively expressed in all developmental stages, but most highly expressed in the adult stage [16]. Similar to the two reports, we found three lepidopteran PP5 genes were expressed in all tested tissues and life stages. Thus we supposed that insect PP5 likely play some essential roles in the physiological activities. Their higher expression in certain stages and in some tissues indicated that they are likely involved in growth development or tissue-dependent activities through interaction with specific regulators or substrates. Further study needs to be done to uncover the physiological roles of insect PP5.

### Expression and Purification of Recombinant PP5 Proteins

Normally, it is difficult to produce active PPP-type phosphatases in *E. coli* mainly due to incorrect folding and lack of the metal ions which are indispensable for phosphatase activity. To facilitate the soluble and active PPP-type expression in *E. coli*, chaperones, such as GroEL/GroES, were co-expressed [17]. In addition, a low concentration of metal ions (usually 1–4 mM MnCl_2_) was externally added to the culture media [18]. Successful expression of active recombinant PP5 in *E. coli* has been reported in mammals [4], *Plasmodium falciparum* [19], and *Trypanosoma brucei* [20]. Recently, we obtained active *E. coli*-yielded recombinant PP5 from *P. xylostella* and the cantharidin-producing beetle, *E. chinensis* [12], [16]. No other studies have focused on recombinant PP5 in other insects.

**Figure 4:**
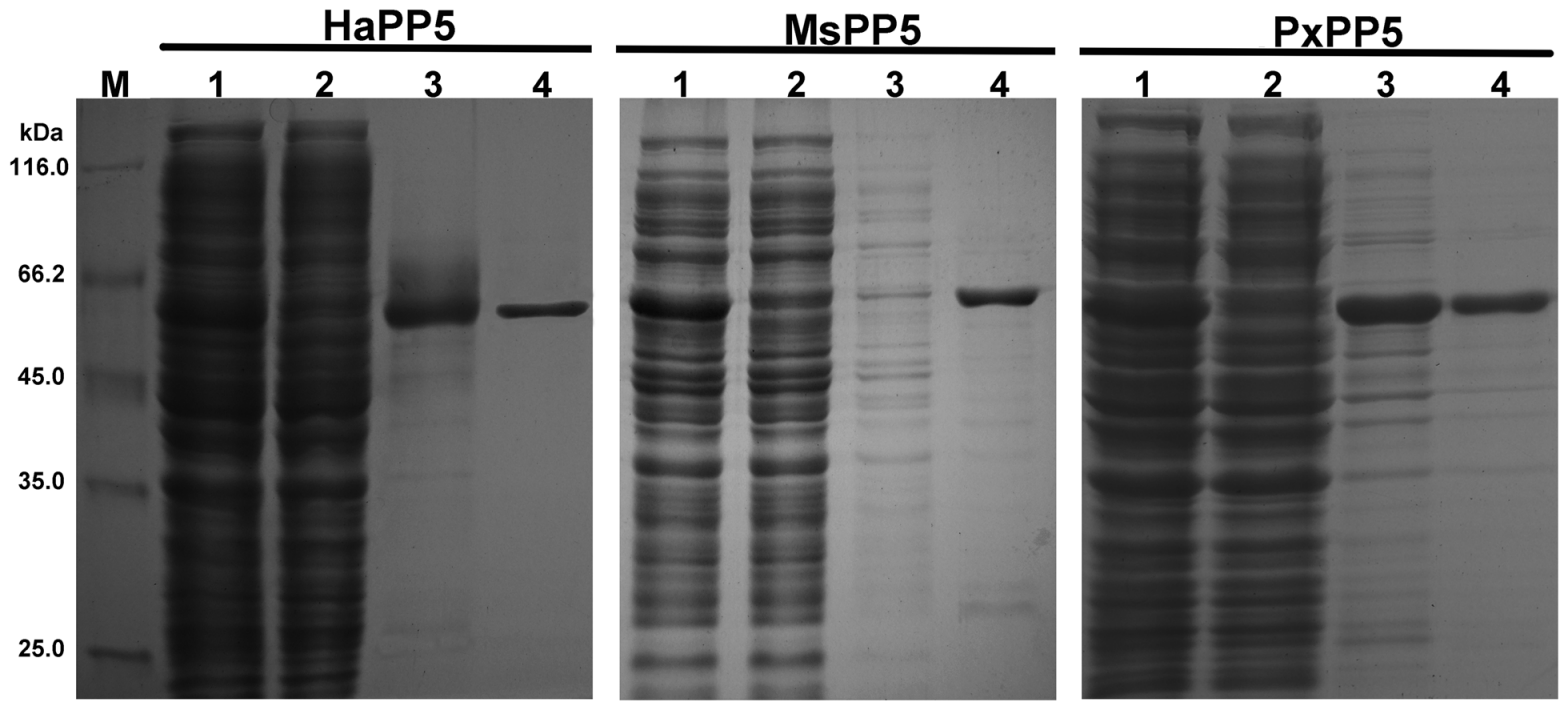
Purification of *E. coli* produced recombinant PP5 proteins. Lane M: protein marker; Lane 1: total soluble cell lysate; Lane 2: flow-through elution; Lane 3: wash-down elution; Lane 4∶150 mM imidazole elution.

To compare the *in vitro* characterization of recombinant PxPP5, HaPP5, and MsPP5, their ORFs were all subcloned into the pET28a (+) vector. Recombinant proteins were produced and purified under the same conditions. 12% SDS-PAGE analysis showed that three recombinant PP5 were soluble expressed and successfully purified with bands of ∼60 kDa being close to their predicted protein sizes. Relatively high purity recombinant PP5 proteins (∼90%) were eluted by 150 mM imidazole (Fig. 4). The elutions were pooled and dialyzed overnight at 4°C, then stored at −20°C. Finally, 2.4 mg, 2.8 mg, and 3.4 mg of recombinant HaPP5, MsPP5, and PxPP5 proteins were obtained from 500 ml LB media.

### Biochemical Characterization of Recombinant PP5 Proteins

It is known that both native and recombinant PP5 proteins display low levels of basal phosphatase activity [4], which is principally due to its N-terminal domain folding over the catalytic site blocking access to substrate [5], [21]. However, polyunsaturated, long-chain fatty acids such as arachidonic acid (AA) could stimulate PP5 activity by directly interacting with the TPR domain to alter the conformation of the PP5 TPR domain which presumably results in the release of autoinhibition [5].

**Figure 5:**
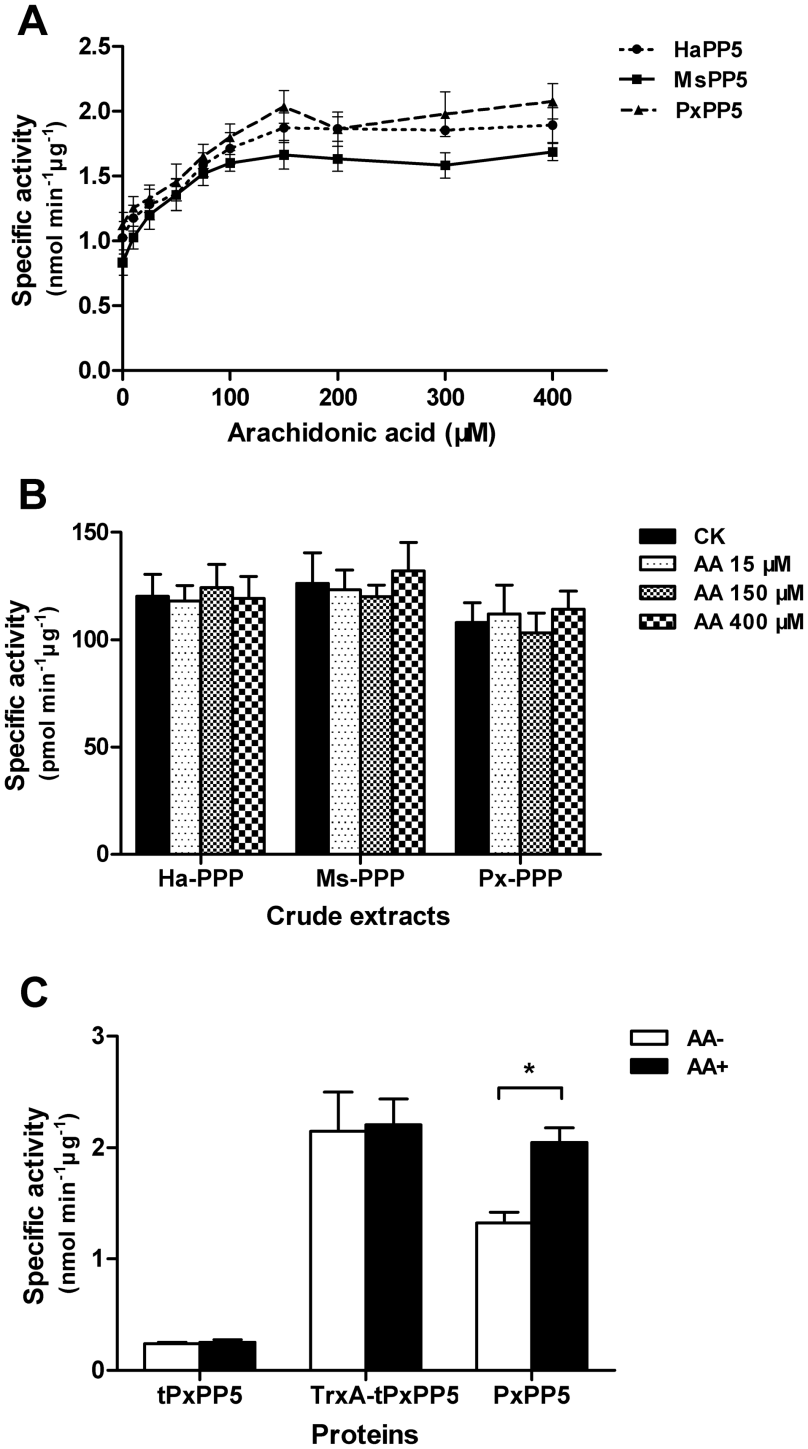
Arachidonic acid stimulation assays. (A) Arachidonic acid stimulation of recombinant HaPP5, MsPP5, and PxPP5 proteins. (B) Responses of crude PPP extracts to arachidonic acid using phosphopeptides as substrate. (C) Responses of tPxPP5 (TPR-domains truncated PxPP5), TrxA-tPxPP5 (TPR-domains truncated PxPP5 fused with TrxA tag at N-terminus), and PxPP5 (wild type PxPP5) to 150 µM arachidonic acid. Asterisk indicates a significant difference between two groups (*, *p*<0.05) obtained by Student *t*-test. Results are presented as the mean ± SD (n = 3).

The recombinant lepidopteran PP5 proteins were found to possess phosphatase activity toward pNPP. Arachidonic acid stimulated the activity of recombinant HaPP5, MsPP5, and PxPP5 up to 1.78-, 1.99-, and 1.81-fold, respectively, in a dose dependent manner with the maximum activation for all occurring at 150 µM (Fig. 5A). Our results indicated that the enzyme activities of three recombinant lepidopteran PP5 proteins were also modulated by the TPR domain. Previous work reported that the maximum activation of arachidonic acid on *E. coli* expressed PP5 from *T. brucei*, *P. falciparum* and *E. chinensis* were 2.6-, 2.0-, and 2.3-fold, respectively [16], [19], [20]. Whereas, the activity of both *E. coli*-expressed and native mammalian PP5 was low and could be enhanced by arachidonic acid up to much higher degrees [4], [18]. These reports imply that the basal activity of some recombinant PP5 proteins are relatively high, which might due to the changes in conformation when expressed in *E. coli*. The increased intrinsic activity of recombinant PP5 proteins might due to the changes in conformation when expressed in *E. coli*. Purifying the native lepidopteran PP5 to investigate whether arachidonic acid could stimulate their activity to higher folds is challenging. Herein, we conducted the arachidonic acid stimulation assay against total crude protein phosphatases extracted from the larvae of *H. armigera*, *M. separata* and *P. xylostella* using phosphopeptides as substrate. Total extracts showed phosphatase activity, whereas no significant stimulation by arachidonic acid was observed (Fig. 5B), which is probably due to the low abundance of PP5 protein or the activity has been enhanced by an intrinsic activator.

Removal of the N-terminal TPR sequences increased PP5 phosphatase activity [4], [19]. To test whether the absence of TPR-domain could result in an increased ability of lepidopteran PP5 for substrate binding, we removed the N-terminal TPR sequences from the full-length clone corresponding to the first 195 amino acids of PxPP5 and produced a deletion mutant protein tPxPP5 on a pET28a (+) vector. Whereas, unlike the N-terminal lacking fragments of human and *P. falciparum* PP5 proteins where the same levels of phosphatase activity were detected as for the full-length proteins [4], [19], the phosphatase activity of tPxPP5 protein was only 12.23% of the full-length PxPP5 (Fig. 5C). Our finding indicated that incorrect-folding occurred when the phosphatase segment was expressed in *E. coli*, which also implied that TPR domain might play key roles in facilitating the folding of its following phosphatase domain. To investigate whether the low activity of tPxPP5 is due to incorrect-folding in the *E. coli* expression system, the pET32a (+) vector harboring the thioredoxin protein to facilitate the correct-folding of heterologous protein was used to produce the TrxA-tPxPP5 protein. TrxA-tPxPP5 protein showed similar activity with the arachidonic acid activated full-length PxPP5 (Fig. 5C), which indicated that the tPxPP5 segment it contains was well-folded. It seems that, unlike the intrinsic TPR-domain, the fused TrxA segment might not block substrate access to active site of tPxPP5 segment, however, it could be capable of facilitating the correct folding of the tPxPP5 segment.

**Figure 6:**
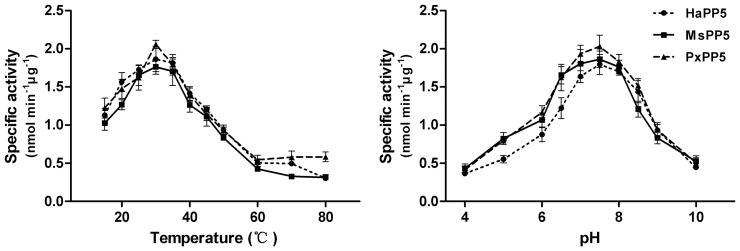
Optimum reaction temperature and pH determination of recombinant PP5 proteins. Results are presented as the mean ± SD (n = 3).

The optimal reaction pH or temperature was determined from a series of reactions in different pH assaying buffer or in pH 7.4 assaying buffer at varying temperatures. The phosphatase activity of all three recombinant PP5 first increased and then declined with ascending pH or temperature. The optimum reaction temperature is around 30°C for these three proteins and the optimum reaction pH is about 7.5 (Fig. 6).

To determine the kinetic properties of three recombinant lepidopteran PP5 proteins, enzyme-substrate reactions were conducted using pNPP as substrate. Kinetic parameters were obtained from Michaelis-Menten plots using GraphPad Prism 5 and are listed in Table 2. In the absence of activator, there was no significant difference among the kinetic values of these three proteins. With the addition of 150 µM arachidonic acid, the *Km* values of the three proteins, HaPP5, MsPP5, and PxPP5, were reduced by 2.07-, 1.86-, and 2.16-fold, respectively, while their corresponding *Vmax* values were elevated by 1.33-, 1.54-, and 1.26-fold, respectively (Table 2). Our result showed that the effects of arachidonic acid on phosphatase activity of lepidopteran PP5 proteins are primarily due to effects on *Km* values. The changes of kinetic values, which are in agreement with reports on human PP5 [6], indicated more of the substrate enters into the active site. This finding also explains that arachidonic acid can interact with the TPR domain of lepidopteran PP5 proteins to release the autoinhibition. Moreover, that these three lepidopteran PP5 proteins share similar kinetic properties is consistent with the high identity of their amino acid sequences.

**Table 2:** Kinetic parameters for p-Nitrophenol production.

Enzyme	Arachidonic acid	*Km*	*Vmax*
	(150 µM)	(mM)	(nmol min^−1 ^µg^−1^)
HaPP5	–	8.64±0.95	1.6±0.16
	+	4.17±0.52	2.12±0.43
MsPP5	–	9.01±0.51	1.34±0.35
	+	4.84±0.12	2.07±0.22
PxPP5	–	8.83±0.75	1.76±0.09
	+	4.09±0.32	2.22±0.31

### Inhibition Study

**Figure 7:**
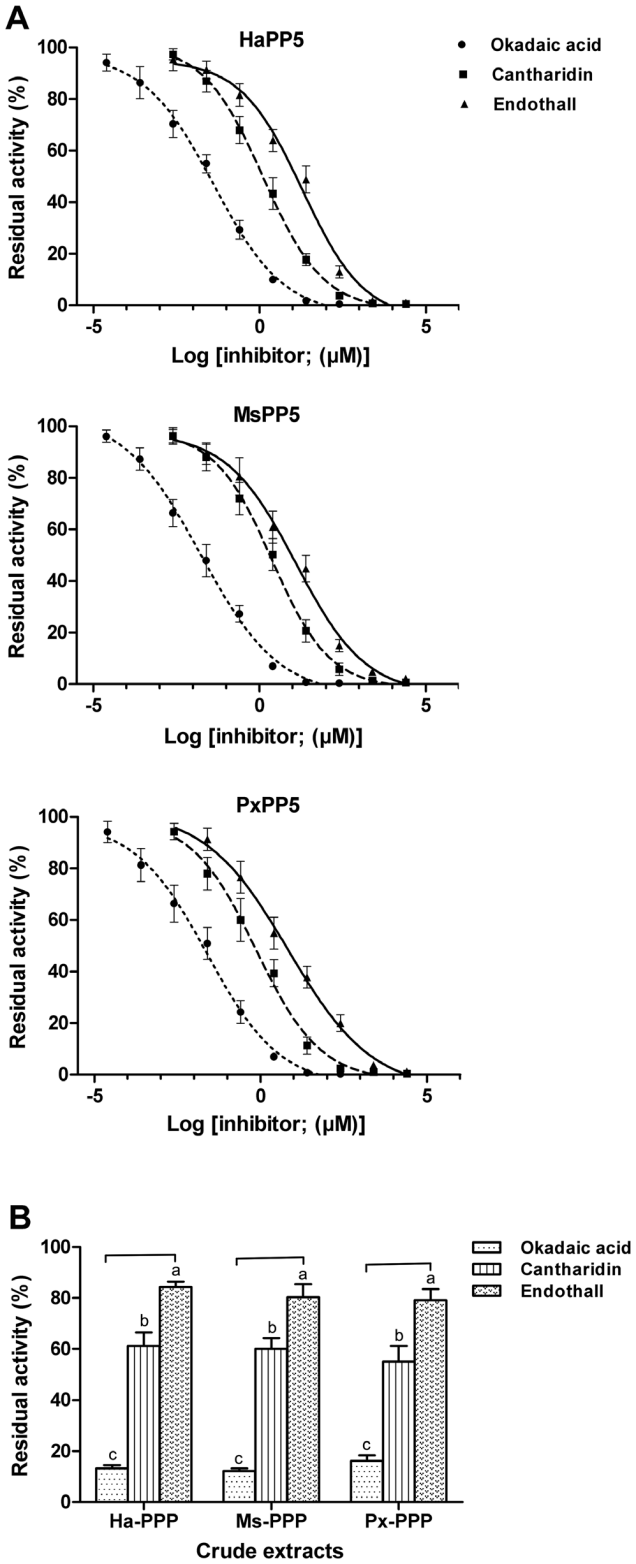
Inhibition assay of protein phosphatase inhibitors against recombinant proteins (A) and crude PPP extracts (B). Results are presented as the mean ± SD (n = 3). The statistic differences were tested by One-Way ANOVA.

Some naturally occurring toxins, such as microcystin-LR, calyculin, okadaic acid, cantharidin, etc., have been found to potently impede the phosphatase activity of both native and recombinant mammalian PP1, PP2A, PP4 and PP5 *in vitro* [22]. To test the sensitivity of recombinant lepidopteran PP5 proteins to phosphatase inhibitors, okadaic acid, cantharidin and endothall (a commercial herbicide) were recruited in the inhibition assay. As expected, they impaired the activities of recombinant PP5 in a dose-dependent response (Fig. 7A). The IC_50_ values of the three inhibitors on three recombinant lepidopteran PP5 proteins are listed in Table 3.

**Table 3:** IC_50_ values of inhibitors against recombinant PP5 proteins.

	Inhibition of phosphatase activity (IC_50_, µM)		
Compound	HaPP5	MsPP5	PxPP5
okadaic acid	41.82±3.31 nM	17.57±3.20 nM	25.01±1.64 nM
cantharidin	1.28±0.31	2.38±0.48	0.73±0.27
endothall	19.33±2.34	12.64±1.62	6.84±0.47

In general, the three proteins showed a similar level of sensitivity to each inhibitor and the inhibiting ability of the three inhibitors against lepidopteran PP5 proteins ranks as: okadaic acid>cantharidin>endothall. The IC_50_ values of okadaic acid on lepidopteran PP5 proteins (17.57 nM on MsPP5, 25.01 nM on PxPP5, and 41.82 nM on HaPP5) are significantly higher than those reported for mammalian PP5 (IC50 = 3.5 nM), but are much lower than in *E. chinensis* PP5 (IC_50_ = 190.0 nM) [16]. Cantharidin impeded the activities of three lepidopteran PP5 proteins to a similar degree with IC_50_ values of 0.73–2.38 µM which are consistent with previous reports on other sources of PP5 [12], [16], [22]. The IC_50_ values of endothall were 6.84 µM for PxPP5, 12.64 µM for MsPP5, and 19.33 µM for HaPP5, respectively. According to our results, compared to the other two proteins, MsPP5 was more sensitive to okadaic acid, while cantharidin and endothall blocked PxPP5 activity more potently than they did the other two proteins. These findings indicated that although lepidopteran PP5s possess high sequence similarity and identical key residues, other residues might be involved in the inhibitor binding as well as the conformational differences that may also exist among them.

Members of the PPP family are highly conserved at their phosphatase domains. It has been reported that cantharidin and endothall impaired the phosphatase activity of PPP-extract from *Arabidopsis thaliana*, *Lemna paucicostata* and *E. chinensis* [16], [23]. Here we found these three compounds showed an inhibitory effect on PPP activity in the crude extracts of three lepidopteran insects (Fig. 7B). The residual phosphatase activities of the three crude extracts treated with 1.0 µM okadaic acid, cantharidin, and endothall were 16.37–18.89%, 57.65–61.23%, and 83.77–87.66%, respectively. This result is in agreement with the above result of their inhibitory effect on recombinant PP5 proteins indicating that other members of PPP are also sensitive to these inhibitors. Our results also provide a view that PPP inhibitors might be utilized in lepidopteran pest control.

Specific inhibitors have been proved to be valuable tools to explore the physiological processes of target proteins. Changes in multiple cellular pathways at their transcription levels were observed in plant and in mammals post cantharidin treatment indicating the involvement of PPP members in these pathways [24], [25]. These three phosphatase-specific inhibitors could be used to investigate the physiological roles of the PPP family in lepidopteran and other insects in further study. As for the determination of the function of any single PPP member, such as PP5, more specific inhibitors should be used. Determining the biological roles of PP5 has proven challenging. Since there is no PP5-speicific inhibitor that has been developed, suppressing the expression of insect PP5 with antisense oligonucleotides or siRNA would be a promising alternative to reveal their unique functions.

## Conclusions

Many lepidopteran insects are of great economic importance because many of them are agricultural pests. We identified cDNAs of HaPP5, MsPP5, and PxPP5. Analyses of their expression patterns revealed relatively high transcript abundance in egg and adult stages. Biologically active recombinant proteins were produced in *E. coli* with similar biochemical properties. Three protein phosphatase inhibitors showed a strong inhibitory effect on both recombinant proteins and crude protein phosphatase extracts suggesting their potential utilization value for lepidopteran pest control.
